# A Machine Condition Monitoring Framework Using Compressed Signal Processing

**DOI:** 10.3390/s20010319

**Published:** 2020-01-06

**Authors:** Meenu Rani, Sanjay Dhok, Raghavendra Deshmukh

**Affiliations:** Centre for VLSI and Nanotechnology, VNIT Nagpur, Nagpur 440010, India; sbdhok@ece.vnit.ac.in (S.D.); rbdeshmukh@ece.vnit.ac.in (R.D.)

**Keywords:** compressive sensing, compressed signal processing, bearing fault diagnosis, feature extraction, random demodulator

## Abstract

The vibration monitoring of ball bearings of a rotating machinery is a crucial aspect for smooth functioning and sustainability of plants. The wireless vibration monitoring using conventional Nyquist sampling techniques is costly in terms of power consumption, as it generates lots of data that need to be processed. To overcome this issue, compressive sensing (CS) can be employed, which directly acquires the signal in compressed form and hence reduces power consumption. The compressive measurements so generated can easily be transmitted to the base station and the original signal can be recovered there using CS reconstruction algorithms to diagnose the faults. However, the CS reconstruction is very costly in terms of computational time and power. Hence, this conventional CS framework is not suitable for diagnosing the machinery faults in real time. In this paper, a bearing condition monitoring framework is presented based on compressed signal processing (CSP). The CSP is a newer research area of CS, in which inference problems are solved without reconstructing the original signal back from compressive measurements. By omitting the reconstruction efforts, the proposed method significantly improves the time and power cost. This leads to faster processing of compressive measurements for solving the required inference problems for machinery condition monitoring. This gives a way to diagnose the machinery faults in real-time. A comparison of proposed scheme with the conventional method shows that the proposed scheme lowers the computational efforts while simultaneously achieving the comparable fault classification accuracy.

## 1. Introduction

The industrial plants consist of several types of machines. The rotating parts in these machines are often subjected to mechanical tear and wear [1]. If the attention is not paid towards this wear and tear, it may lead to the breakdown in the machines and unexpected shutdown in the plant [2]. Apart from mechanical faults, machines can also develop electrical faults, which may be the reason behind serious hazards. Therefore, condition monitoring of these machines is very important for early stage fault detection. This avoids unscheduled repairs, minimize failures and hence, guarantee reliability, availability, and sustainability of machines, which saves time, money, and power consumption [3]. Several noninvasive machine condition monitoring techniques have been proposed in literature. These techniques are based on measuring either electrical quantities like current, voltage, and external magnetic field, or the mechanical quantities like acceleration, velocity, force, etc. A review of the emerging machine condition monitoring techniques has been presented in [4]. The most commonly used technique is the motor current signature analysis (MCSA), which is based on analyzing the spectrum of the stator current signal. But this method works fine if the machine is operating at constant speed and rated load. For the faults induced due to rotor eccentricities, phase current monitoring is an easy way. Split phase current signature analysis (SPCSA) is another method, which utilizes the air gap flux density modulation due to rotor eccentricities. Recently, methods based on stray flux measurements have been proposed. The methods are noninvasive and simple to implement but are difficult to model [5]. Another recent noninvasive approach for real-time fault diagnosis is the Parks’ vector approach, which makes use of inexpensive sensors and measurement system [4].

To diagnose the gear and bearing faults, vibration sensors are installed on the machines to monitor their vibration trends. Typically, faults in the ball bearings of machine are detected with the help of vibration monitoring of machines [6]. The vibration data may be obtained by measuring displacement, velocity, acceleration or force, which can be processed to determine the type and severity of fault, if present [7]. Several digital signal processing (DSP) techniques can be used to process the vibration data like fast Fourier transform, discrete Fourier transform, short-time Fourier transform, wavelet transform, etc. These techniques are cost-effective and can be implemented for real-time fault diagnosis. utilizing these advantages of DSP techniques, an online method for early stage bearing fault diagnosis has been presented in [8,9]. These techniques are based on decomposing the vibration signal sub-bands using wavelet transform.

In this era of information age, the machinery vibrations are monitored remotely through wireless links. For this, the battery operated wireless sensor nodes are installed on the machines. These nodes continuously sense the machinery vibrations and send these data to the base station. At the base station these data are processed and the machinery condition is identified. As these nodes generate a huge amount of data, therefore the major hurdle in wireless vibration monitoring is the handling of huge amount of data with power constrained nodes. To reduce the transmission power, the data sensed with traditional sampling mechanism require compression before transmission to the base station. The compression stage drains a lot of battery power [10,11]. Therefore, traditional sampling mechanism overburdens the sensor node. In this scenario, compressive sensing (CS) performs superior to the traditional method. CS is a relatively newer sensing method, which samples at a much lower rate than traditional method. This in turn generates far fewer samples and hence reduce the power required for sampling, compression and transmission [12,13].

The mathematical background of CS was developed by Donoho, et al. in 2004 [14,15]. CS has been shown to be applicable for the signals which are either sparse or compressible, either in their original domain or in some transformed domain. Since, almost all the signals have sparse or compressible representation in some domain, therefore, applicability of CS extends to a wider range of signals [16]. The speciality of CS is that the sampling rate in case of CS is decided by the signal sparsity as compared to the Nyquist-criterion, where the sampling rate depends upon the highest frequency component present in signal. CS works by the mechanism of random sampling. Therefore, the CS measurements tend to be non-adaptive, i.e., current measurement has no relation with the previous measurements [17]. Another quality of CS is that the original signal can be reconstructed faithfully from these random measurements with the help of some non-linear technique, e.g., convex optimization or some iterative methods, subject to the constraints of signal sparsity, minimum number of compressive measurements required, incoherence between sensing matrix and signal sparsifying basis [18]. The major applications of CS are in the fields, like: non-visible wavelengths acquisition, imaging via neutron scattering, medical imaging, ultra wideband signal acquisition, etc. [19,20].

**Figure 1:**
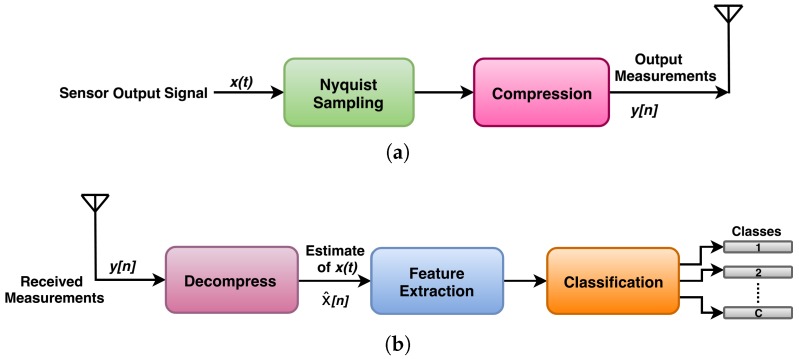
Scenario representing WSN implementation using Nyquist sampling technique: (**a**) sensor node and (**b**) base station.

**Figure 2:**
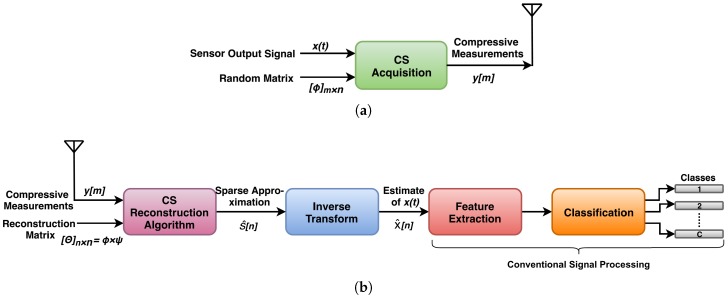
Scenario representing WSN implementation using conventional CS technique: (**a**) sensor node and (**b**) base station.

A precise and timely diagnosis of machinery faults is the crucial aspect of condition monitoring. In this regard, the Nyquist and conventional CS frameworks for remotely monitoring the machinery health are shown in Figure 1 and Figure 2. The signal acquisition using Nyquist framework is done via oversampling the signal by many orders of magnitude greater than Nyquist rate. This generates too many samples. After compression, these samples are transmitted to the base station. These stages consumes a lot of power. At the base station, which is not power constrained, the received signal is decompressed to obtain the estimate of original signal. Then the relevant features are extracted from the signal to identify its class. In conventional CS framework, the CS acquisition is first implemented on remote node and then the compressive measurements are transmitted to the base station. At the base station, the vibration signal is reconstructed from compressive measurements to extract the relevant features for diagnosing the faults using traditional signal processing techniques. Both of the above mentioned frameworks are not suitable for the real time fault diagnosis because: (i). the Nyquist framework generates too many samples which consumes a lot of power for processing. (ii). conventional CS uses complex reconstruction and cannot be implemented on node. For real time monitoring the major requirement is that the relevant features extraction and their classification must be done on node within the available power budget [21,22].

**Figure 3:**
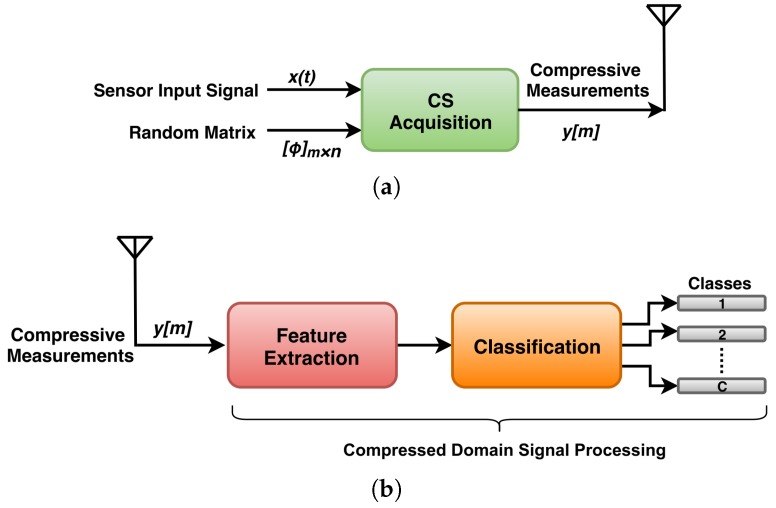
Scenario representing WSN implementation using CSP technique: (**a**) sensor node and (**b**) base station.

In this paper, a bearing condition monitoring framework is presented based on compressed signal processing (CSP). CSP is a newer research area of CS, in which inference problems are solved without reconstructing the original signal back from compressive measurements. The concept of CSP is based on the fact that the compressive measurements carry sufficient information about the original signal, from which it is possible to recover the original signal back. Apart from recovering, the other signal processing tasks like: classification, detection and estimation, can also be performed on compressive measurements [23,24,25]. A scenario representing WSN implementation using CSP is shown in Figure 3. Here, the implementation of the sensor node is same as that in the conventional CS scenario, while the difference lies in the implementation of base station. In this, using some modified signal processing technique, the features are directly extracted from the compressive measurements, instead of first recovering the original signal. These features are then further used for classifying the condition of the system under supervision. CSP enables the real time machine condition monitoring and early stage detection of faults to avoid the unexpected plant shutdowns [26,27]. The proposed scheme for bearing condition monitoring is based on CSP and is applied on the bearing vibration database taken from laboratory for dynamics of machines and structures (Ladisk) [28]. The authors of [28] are associated with this laboratory and they have prepared this vibration dataset by measuring the reactive forces due to vibration excitation. This dataset is freely available to use. For extracting the features directly from compressive measurements of bearing vibration signal, a feature extraction method has been proposed. Then, for classifying these features, several classifiers have been tested and the best one is used for classification purpose.

The further organization of this paper is as follows: Section 2 discusses the background and related work in this area. Section 3 describes the proposed framework for bearing condition monitoring based on CSP. Section 4 presents the supporting results and related discussion.

## 2. Background and Related Work

In this section, the background related to generating compressive measurements and work related to the bearing fault diagnosis from compressive measurements are presented. Let, $x\in R^{n}$ or $C^{n}$ be an input signal, $\varphi \in R^{m\times n}$ or $C^{m\times n}$ be a random measurement matrix and $y\in R^{m}$ or $C^{m}$ be the output measurement vector, where m≪n. The mathematical model for generating CS measurements corresponding to an input signal is given by (1) [20].

(1) y=φx,

For gathering sufficient information about the input signal, the minimum number of CS measurements to be taken depends upon the sparsity of underlying signal. Their number can be further reduced by introducing incoherence between measurement and sparsifying basis of signal, where sparsifying basis is the basis in which signal has sparse representation [19]. CS basically uses random matrices as the measurement basis, which are incoherent with any other basis. For perfect reconstruction, a relation between minimum number of measurements, *m*, sparsity, *k* and coherence, μ is given by (2) [7,29].

(2) $$m\geq c\mu^{2}k\log n.$$

Although the original signal can always be reconstructed back from compressive measurements subject to the constraints of RIP and incoherence, but this conventional CS framework is not suitable for real time fault diagnosis due to high reconstruction cost. The major requirement of self diagnosing systems is that the processing should be done on node for faster and accurate diagnosis. In this scenario, the framework based on CSP seems to fit well, because of its inherent advantage of omitting the need of reconstruction. This directly overcomes the reconstruction cost and also allows to relax the constraint of minimum number of compressive measurements imposed by signal recovery. This means that more and more undersampling can be done till the inference problem can be solved with sufficiently high accuracy, without bothering about the signal recovery [27]. The related work concerning the bearing fault diagnosis directly from compressive measurements includes: bearing fault detection by incomplete reconstruction, in which reconstruction process is terminated in-between, once the harmonics related to fault are identified [30]. Although, this method saves power by only solving the incomplete reconstruction, but this also cost some power, hence is not suitable in power constrained environment. Another approach suggested by Zhang et al., for bearing vibration monitoring from compressive measurements is using several trained dictionaries corresponding to the bearing faults, for diagnosing the faults from compressive measurements [31]. This method is computationally expensive, because corresponding to each and every fault, the trained dictionaries need to be stored and the compressive measurements need to be sparsified against each and every dictionary to identify the dictionary for which sparsest representation is obtained. The dictionary so identified corresponds to the fault. As this method is computationally expensive, hence it is not suitable for the purpose targeted in this paper. Another method proposed by Shao et al., is using the concept of convolutional deep belief network along with Gaussian visible units for learning the features from compressive measurements [32]. Their method also increases the computational burden and hence, costs power. In contrast to the above methods, the proposed method is simpler and less computationally expensive and can support the machinery fault diagnosis in real time.

## 3. Proposed Framework

**Figure 4:**
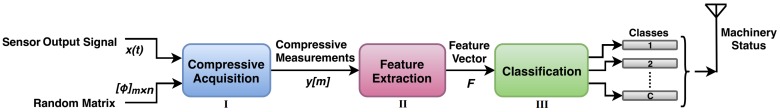
The proposed machinery condition monitoring scheme based on compressed signal processing (CSP) for real time fault detection.

The proposed framework for machinery condition monitoring is shown in Figure 4. This consists of the following stages: (i) compressive acquisition stage, (ii) feature extraction stage, and (iii) classification stage. In the first stage, the signal acquisition using CS is performed to generate compressive measurements. The second stage processes these measurements to extract relevant features. The final stage uses a trained classifier to classify the features into different classes. The details of these stages are presented below:

### 3.1. Stage-I: Compressive Acquisition

**Figure 5:**
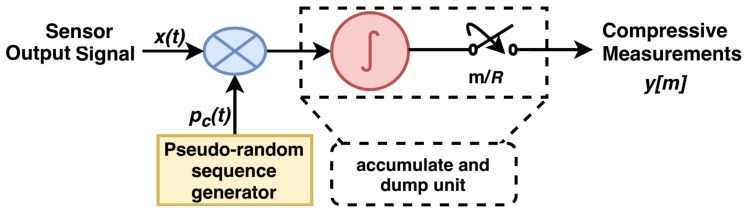
Stage-I: compressive acquisition of sensor output signal using random demodulator.

This first stage uses a CS acquisition method to sense the input signal. The various CS architectures are available in literature, e.g., random demodulator (RD) [33], random modulator pre-integrator (RMPI) [34], compressive multiplexer (CMUX) [35], etc., for this purpose. In this paper, RD has been used for the acquisition of bearing vibration signals, because of its simple architecture. RD was proposed by Laska et al., in 2007 and is an efficient wide-band signal sampler. It can sample signals at a rate much below the Nyquist. The architecture of RD is shown in Figure 5. Here, the input signal *x(t)* is first randomized by multiplying with a pseudorandom noise like sequence, say of +/−1s. This sequence is called chipping sequence $p_{c}\left(t\right)$. This randomization spreads the signal frequencies to lower as well higher frequency regions. The resulted signal is then passed through an integrator, which accumulates the multiplied signal for a specific duration and generates its unique frequency signature occupying the lower frequency region. This low frequency signal is then sampled at a rate much below the Nyquist rate of original signal and hence, results in fewer compressive measurements. These measurements give a unique frequency signature corresponding to a particular input signal frequency and hence, preserves the information about the original signal. This information is useful in recovering the original signal back from compressive measurements, as well as can be used for inference applications.

The matrix form of RD operation is governed by (3) and (4). Here, *P* consists of n−terms of chipping sequence along the diagonal. The multiplication with this matrix randomizes the input signal, represented by $\tilde{x}$. The matrix *H* serves the purpose of accumulate and dump unit. This is an m×n matrix, responsible for undersampling the signal by accumulating a specific number of samples of $\tilde{x}$. The number of samples to be accumulated for generating a single output measurement is given by the R=⌊n/m⌋, which is the number of ones in single row of *H*. The product of two matrices *P* and *H* mimics the random matrix, φ [33,36].

(3) $$P=\left[\begin{array}{ccc} p_{1}\\ \; & \ddots \\ \; & \; & p_{n} \end{array}\right];\;H=\left[\begin{array}{ccc} 111\cdots \\ \; & 111\cdots \\ \; & \; & 111\cdots \end{array}\right]$$

(4) $$\left.\begin{array}{ccc} \tilde{x}= & Px & \\ y= & H\tilde{x}= & \varphi x\\ \varphi = & HP & \end{array}\right\}.$$

### 3.2. Stage-II: Feature Extraction

After obtaining compressive measurements, the second stage is the feature extraction stage. As compressive measurements preserve the structure of underlying signal, which means that the energy of compressive measurements will be specific for a specific input signal. Here, the energy in compressive measurements of bearing vibration signal is used as a feature to distinguish between the different conditions of a bearing. The features are extracted from compressive measurements for the different undersampling factors under consideration. For comparison purpose, the features are extracted from original signal as well. The process of feature extraction from original signal and from compressive measurements is described below. In order to use the same filter coefficient matrices, a slight modification in feature extraction process is required in case of compressive measurements compared to the original signal.

#### 3.2.1. Feature Extraction from Original Signal

**Figure 6:**
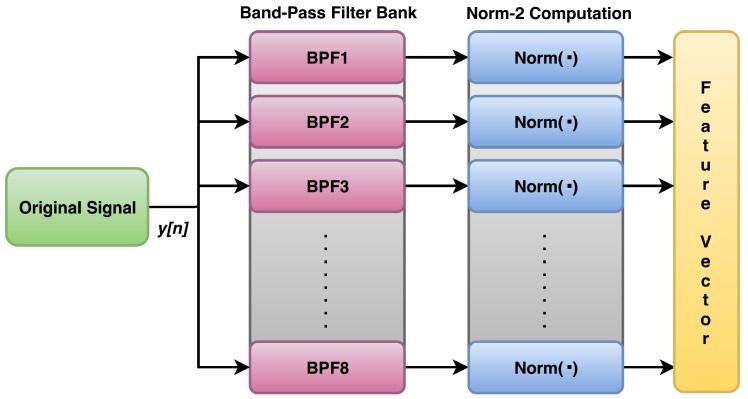
Process of feature extraction from Nyquist samples.

For easing the feature extraction process, the signal is first divided into segments. To use energy as a feature, the energy from each segment of original signal is extracted with the help of band-pass filters and norm-2 computation, as shown in Figure 6. Here, each signal segment is passed through a bank of eight band-pass filters (BPFs), which divides the signal bandwidth into eight equal bands. The reason for using eight filters for each segment is that it improves the fineness in features, which enhances the distinguishability among features belonging different classes. Use of more number of band-pass filters will not significantly improve the fineness in features but will further increase the computational burden. After this, the energy of these filtered signal segments is extracted by computing the 2-norm of each filtered output. This generates a single feature of dimensionality eight. Similarly, the other elements of feature vector are generated from the next segments of bearing vibration signal.

#### 3.2.2. Feature Extraction from Compressive Measurements

**Figure 7:**
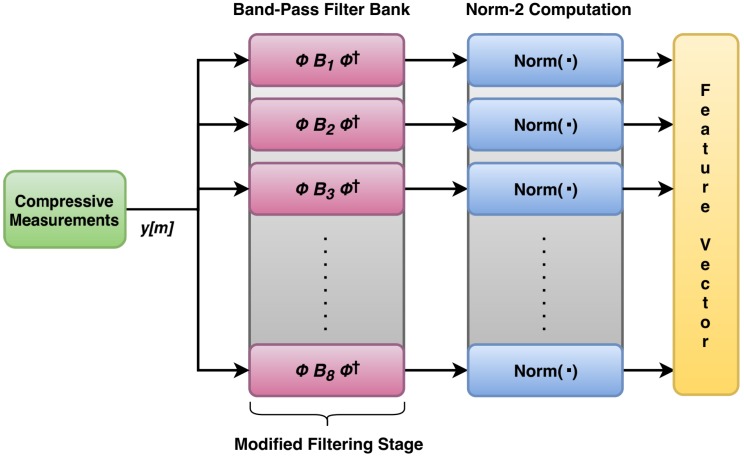
Method used for extracting features from compressive measurements.

Here also, the compressive measurements are first generated segment wise and for each segment, the features are extracted. The process of feature extraction from compressive measurements is shown in Figure 7. In order to use the same filter matrices, which were designed for original signal, a slight modification in above filtering process needs to be done for extracting the features from compressive measurements. Here, the filter coefficients matrix $B_{i}$ is modified by pre-multiplication with φ and post-multiplication with $\varphi_{R}^{†}$, as Equation (5):

(5) $${\hat{B}}_{i}=\varphi B_{i}\varphi_{R}^{†}.$$

This generates m×m filter matrices having dimensions compatible with the compressive measurements and hence can be easily used with these measurements. The mathematical derivation of this formulation is presented below by assuming $z_{i}$ be the output of $i_{th}$ conventional filter and ${\hat{z}}_{i}$ be the output of $i_{th}$ modified filter, then ${\hat{z}}_{i}$ is obtained by randomizing the $z_{i}$ as:

$${\hat{z}}_{i}=\varphi z_{i}$$

$${\hat{B}}_{i}y=\varphi B_{i}x$$

$${\hat{B}}_{i}\varphi x=\varphi B_{i}x$$

$${\hat{B}}_{i}\varphi =\varphi B_{i}$$

post-multiplication with $\varphi^{†}$

$${\hat{B}}_{i}\varphi \varphi^{†}=\varphi B_{i}\varphi^{†}$$

$${\hat{B}}_{i}=\varphi B_{i}\varphi^{†}$$

where, *y* is the set of compressive measurements which are also the input of modified filters here and *x* is the corresponding set of original signal measurements, also the input of conventional filters. This relaxes the need to design m×m filter matrices separately corresponding to each undersampling factor. Another advantage of this design is that compared to using the separate conventional m×m filter matrices, the modified design yields better results when used with compressive measurements. If using conventional m×m filters, then one needs to design separate filters corresponding to different undersampling factors used, while in the modified design, the single n×n filter matrix designed for original signal is used for compressive measurements corresponding to all the CS undersampling factors. The multiplication given in (5) converts the size of n×n filter matrices to m×m according to the undersampling factor used. A comparison of their performance is presented in the next section. Here, the set of compressive measurements are first passed through the modified filter stage, generating eight filtered signals. The energy of each filtered signal is then extracted by computing its 2-norm. This generates a single feature of dimensionality eight. The whole process is repeated for all the segments belonging to respective undersampled signal to obtain the corresponding feature vector.

### 3.3. Stage-III: Classification

**Figure 8:**
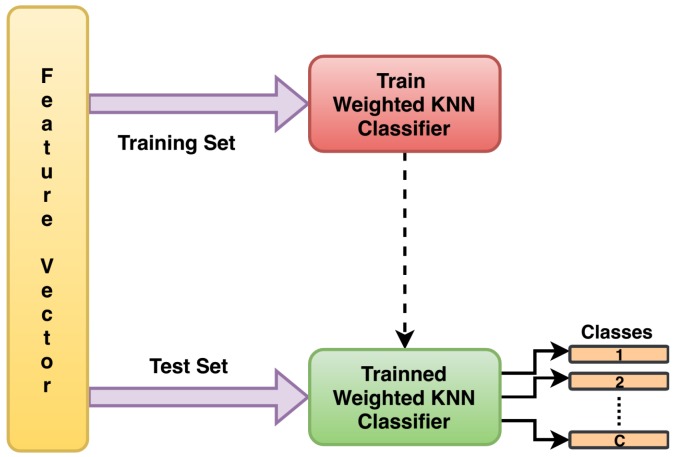
Process of classification of extracted features using weighted *k*-nearest neighbour (*k*-NN) classifier.

In the third stage, classification from the features extracted needs to be done. The classification problem dealt in this paper is a four class classification problem, as the four types of bearing faults have been considered for experimentation. For this purpose, several classifiers like, linear support vector machine (SVM), cubic SVM, fine *k*-nearest neighbour(*k*-NN), weighted *k*-NN, etc. have been trained. The feature set on which classifier training and testing has to be done, is first divided into training and test sets. 50% of the data are used as training set and the remaining 50% are used for testing. The training is done separately on the features extracted from the original signal and from the compressive measurements for different undersampling factors. All the trained classifiers are then tested using different set of feature vectors. The classifier giving highest testing accuracy in almost all the cases is then selected for classification. A comparison of classification accuracies obtained is presented in next section. It has been found that out of these classifiers, the weighted *k*-NN classifier gives highest testing accuracy in almost all the cases, hence is selected for classification purpose in this work. The process of training and classification using weighted *k*-NN classifier is shown in Figure 8.

## 4. Results and Discussion

**Figure 9:**
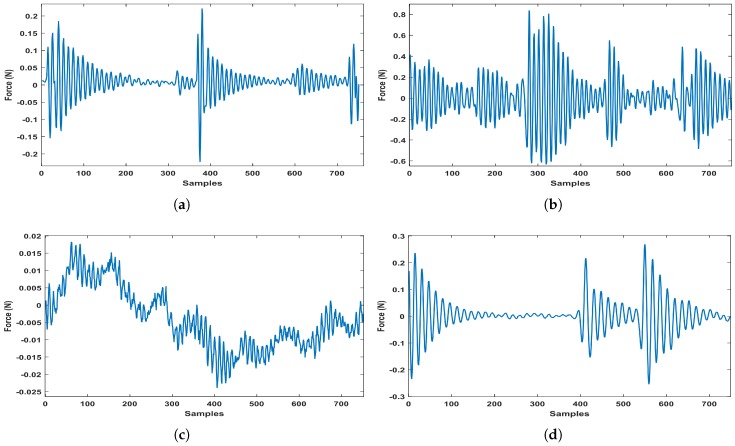
Bearing vibration signal taken from Ladisk: (**a**) signal with axial fault, (**b**) signal with contamination in lubricant fault, (**c**) new bearing vibration signal and (**d**) signal with radial fault.

The bearing vibration data taken from Ladisk are shown in Figure 9 [28]. This database uses force measurements for determining various bearing faults during the assembly process. The database consists of six categories of vibration signal. But for simplicity, the experiments were performed on only four categories of the signal, namely: axial fault, lubricant contamination fault, new bearing signal, and radial fault. For this, a total of 2,400,000 samples of input signal were taken and were processed in the segments of length 500. Each segment was equivalent to 0.1 s epoch of the signal. On these data, all the simulations were done in MATLAB 2017a. For obtaining the features from original signal, eight filter matrices of size 500×500 were obtained from the filter coefficients. These 128 order FIR filters were designed to divide the signal bandwidth into eight equal parts. The frequency of these filters ranged from 10 Hz to 2500 Hz. Therefore, the band of these eight BPFs was divided as: BPF1 is 10–320 Hz, BPF2 is 321–630 Hz, BPF3 is 631–940 Hz, and so on. These bands were not independent as the harmonics of a frequency in a band could be found in higher bands as well. For a single segment of original signal, the filtering by eight band-pass filters generated a single feature vector of dimension 1×8. The energy of this feature vector was extracted by computing its 2-norm as per Figure 6. By repeating this process for all the segments, a feature vector of dimension 4800×8 was obtained. The feature extraction process was same for compressive measurements with a little modification in the filtering stage. This process was repeated for the different undersampling factors under consideration, in case of compressive measurements. For each case, the feature vectors of dimension 4800×8 were obtained, as shown in Table 1.

To show the effectiveness of the modified filtering stage used for feature extraction from compressive measurements, over conventional filters, a comparison is presented in Table 2. If the conventional filters are used then different size filter matrices $B_{ci}$ are required corresponding to different CS undersampling factors, while using the modified filtering, the $B_{i}$ matrices designed for original signal suffice for compressive measurements as well. Only the size of filter matrices need to be converted as per (5) to obtain ${\hat{B}}_{i}$. The comparison of accuracies of these two schemes shows that the scheme using modified filtering stage gives higher accuracy compared to the scheme using conventional *m* size filters.

The classification was done separately for all the feature vectors so obtained using several classifiers. A comparison of accuracies obtained using these classifiers is presented in Table 3. Out of these classifiers, the weighted *k*-NN classifier was observed to give higher accuracy and hence was selected for classification purpose in this work. After dividing the feature vectors into training and test sets, the classifier was trained on the training set using 10-fold cross validation. The performance of trained classifier was then tested on test set. The testing accuracies obtained are shown in Table 1. This table compares the classification accuracies obtained on the features extracted from original Nyquist rate signal with the features extracted from the compressive measurements for different undersampling factors. The accuracy obtained on features extracted from original signal was 99.2%. On the other hand, the proposed method, which directly applied feature extraction and classification on compressive measurements, was able to achieve comparable accuracies for the different undersampling ratios considered here. The gain here is in terms of the power saving achieved by using CS acquisition and by directly extracting features from compressive measurements without reconstructing the original signal. A comparison of computational complexity is also presented in Table 4. This table compares the block-wise computational cost of processing the signal using conventional CS and using the proposed method. Although the cost of acquisition and classification stages are same for the two methods, but our method outperforms in case of communication, reconstruction, inverse transform and feature extraction stages compared to the conventional method. The complexity of reconstruction stage used in conventional method varies with the reconstruction approach used [37]. The complexity of other stages are calculated as per the description given in [38,39]. This table clearly shows the applicability of proposed method for the real time machine condition monitoring.

**Figure 10:**
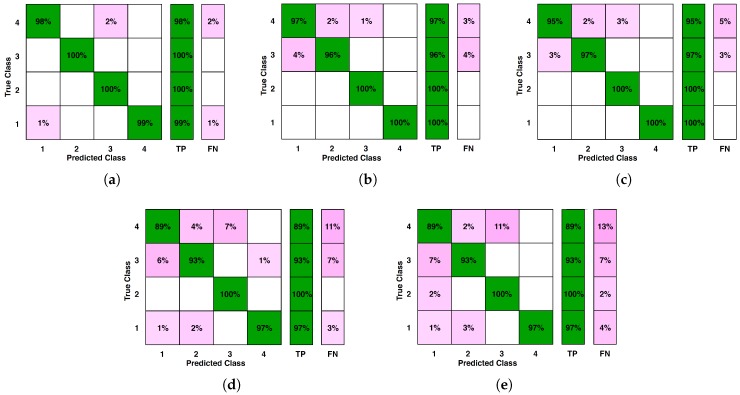
Confusion Matrices of classification results generated by *k*-NN classifier for: (**a**) original signal, (**b**) CS undersampling by factor of 2, (**c**) CS undersampling by factor of 4, (**d**) CS undersampling by factor of 8 and (**e**) CS undersampling by factor of 16.

**Figure 11:**
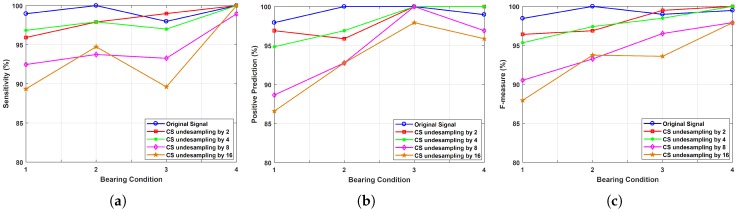
Comparison of classifier performance using: (**a**) sensitivity, (**b**) positive-prediction, and (**c**) F-measure, in classifying the features obtained from original signal and from compressive measurements with varying undersampling ratios for different bearing conditions.

Another performance comparison of the proposed method on this vibration dataset [28] is done using different sensing matrices like: sampling with the matrix of the form [1 1 1 1 0 0 0 0 0 0 … 0 0 0 0 1 1 1 1 0 0 …] (i.e., a non random φ, but a φ with all +1s), (ii) sampling with the matrix of the form [1 0 0 0 0 0 0 0 0 0 …; 0 0 0 0 1 0 0 0 0 …] i.e., the 1:4 subsampling matrix and (iii) sensing with RD matrices [+1 −1 −1 +1 0 0 0 0 0 0 0 0 0 …; 0 0 0 0 −1 +1 +1 −1 0 0 0 0 0 …;]. The results are shown in the Table 5, which shows that RD matrices outperforms the first two cases. Also, the performance of classifier in classifying the features extracted from original signal as well as from compressive measurements is compared using the standard measures i.e., sensitivity, positive prediction and F-measure. These are calculated as per the following equations:

(6) $$\mathrm{Sensitivity}\;\left(S\right)=\frac{TP}{TP+FP}\times 100,$$

(7) $$\mathrm{Positive}\mathrm{prediction}\;\left(P\right)=\frac{TP}{TP+FN}\times 100,$$

(8) $$F\text{-}\mathrm{measure}\;\left(F\right)=\frac{2TP}{2TP+FP+FN}\times 100,$$

where, *TP* stands for true positive, *FP* for false positive and *FN* for false negative. The values of these parameters are computed from the confusion matrices shown in Figure 10. The average results of these three measures for several iterations have been calculated for each condition separately. The range of variations of these measures so obtained have been plotted as shown in Figure 11. This shows that for high undersampling factor like 16, the performance of classifier in classifying the features extracted from compressive measurements is upto the mark and hence, the proposed method performs satisfactorily.

## 5. Challenges and Future Scope

The concept of real time machine condition monitoring is exciting and challenging. Although the techniques based on CSP are efficiently able to overcome the limitations of conventional method in this regard. But still there are some challenges that need to be addressed. Here, some of major challenges in the field are listed along with the associated future scopes.

- The pseudorandom sequence used in the acquisition stage must be good enough in randomizing the input signal. This aspect can be improved to achieve better performance at higher undersampling factors.
- The bearing fault classification is a multiclass classification problem. This requires significant efforts for training and testing the classifiers. So it is very difficult to identify better performing classifier for this purpose. Some efforts can be done to improve upon this part of problem.
- This work can be further extended to test the proposed technique on other bearing vibration datasets.
- The performance of proposed method degrades to some extent at higher undersampling factors. Alternate feature extraction process can be sought for achieving satisfactory performance even at some higher undersampling factors.
- Another future scope of this work is to implement the proposed technique on hardware and analyzing its performance in real time.

## 6. Conclusions

The inherent disadvantages of Nyquist sampling technique discourages its use in power constrained environments like, wireless tele-health monitoring, real time processing, ultrahigh wideband processing, etc. In such environments, the performance of CS has been found to be promising. However, the conventional CS requires signal reconstruction, which is very complex and consumes lot of time and power. So, this framework is not suitable for diagnosing the faults in real time. In this scenario, CSP based approaches are attractive and challenging to work with, because this approach tries to solve the inference problems without reconstructing the original signal back from compressive measurements. This paper proposed an approach based on CSP for monitoring the machinery condition. Here, the features are directly extracted from the compressive measurements and their classification has been done using *k*-NN classifier to diagnose the machinery faults. The performance of proposed method has been compared with the features extracted from original signal and it has been found that the proposed scheme performs satisfactory while reducing the computational complexity to a great extent.

## Figures and Tables

**Table 1:** Training and prediction accuracies of *k*-NN classifier for feature vectors obtained from original signal and from compressive measurements for different undersampling ratios.

Signal Type		Input Signal Dimension	Feature Set Dimension	K-Fold	Testing Accuracy (%)
Original		240,000	4800×8	10	99.2
Compressive measurements for an under-sampling factor of	2	240,000	4800×8	10	98.2
	4	240,000	4800×8	10	97.9
	8	240,000	4800×8	10	94.6
	16	240,000	4800×8	10	93.3

**Table 2:** Performance comparison of scheme using m-size conventional filters and the proposed scheme.

CS Undersampling	Dimensions of Filter Matrices			Classification Accuracy Using	
	Conventional	Nyquist	Modified Filters	Conventional	Modified
	Filters (${B_{c}}_{i}$)	Filter ($B_{i}$)	(${\hat{B}}_{i}=\varphi \times B_{i}\times \varphi^{†}$)	Filters (${B_{c}}_{i}$)	Filters (${\hat{B}}_{i}$)
2	250×250	500×500	250×250	88.7	98.2
4	125×125	500×500	125×125	85.3	97.9
8	62×62	500×500	62×62	79.4	94.6
16	31×31	500×500	31×31	70.8	93.3

**Table 3:** Performance comparison of different classifiers in classifying the features extracted from compressive measurements.

Classifier	Accuracy for CS Undersampling Factor of			
	2	4	8	16
Simple Tree	95.4%	94.6%	89.4%	83.0%
Medium Tree	95.6%	95.6%	93.0%	89.4%
Complex Tree	95.6%	95.6%	93.0%	89.4%
Fine *k*-NN	97.2%	96.4%	94.4%	91.8%
Medium *k*-NN	97.2%	97.2%	94.2%	92.3%
Cosine *k*-NN	95.6%	94.8%	89.4%	87.6%
Cubic *k*-NN	97.2%	97.2%	94.1%	92.0%
Weighted *k*-NN	98.2%	97.9%	94.6%	93.4%
Linear SVM	95.9%	95.4%	93.8%	92.5%
Quadratic SVM	95.6%	95.6%	93.0%	91.5%
Cubic SVM	96.4%	96.1%	93.0%	91.0%

**Table 4:** Comparison of traditional compressed signal (CS) approach and proposed method based on computational complexity

Computational Block	Cost	
	Conventional CS Approach	Proposed Method
CS Acquisition	same	same
Communication	high (transmits measurements)	low (transmits status)
CS Reconstruction (e.g., OMP)	O(n.m.k)	−
Inverse Transform (e.g., IFFT)	O(nlogn)	−
Feature Extraction	high: $O(n^{3})$	low: $O(m^{3})$
classification	same	same

**Table 5:** Performance comparison of proposed scheme using different sampling matrices tested on the vibration dataset taken from Ladisk [28].

CS Undersampling	Classification Accuracy Using		
	Case 1 (Non-Random φ)	Case 2 (Sub-Sampling Matrices)	Case 3 (RD Matrices)
2	91.5%	92.3%	98.2%
4	88.5%	88.7%	97.9%
8	84.0%	85.3%	94.6%
16	79.4%	80.9%	93.3%
